# Draft Genome Sequence of Streptomyces morookaense DSM 40503, an 8-Azaguanine-Producing Strain

**DOI:** 10.1128/MRA.00518-20

**Published:** 2020-07-30

**Authors:** Yueyu Hei, Ziyi Li, Yuhan Zhou, Chenhao Hu, Jin-Ming Gao, Jianzhao Qi

**Affiliations:** a Shaanxi Key Laboratory of Natural Products & Chemical Biology, College of Chemistry & Pharmacy, Northwest A&F University, Yangling, Shaanxi, China; University of Southern California

## Abstract

Here, we describe the genome of Streptomyces morookaense DSM 40503, an 8-azaguanine-producing strain. The genome is the basis for future study and presents an underexplored taxonomy and biosynthetic potential, which expands our understanding of the diversity of microorganisms that produce nitrogen heterocyclic compounds.

## ANNOUNCEMENT

The compound 8-azaguanine is an important natural guanine nucleoside analog. Naturally derived 8-azaguanine was initially reported as an antifungal compound (1). Subsequent studies revealed that 8-azaguanine has significant anticancer effects (2) and can inhibit protein synthesis (3), making it a drug candidate for early anticancer therapy (4). Streptomyces morookaense DSM 40503 is a type strain stored in the German Collection of Microorganisms and Cell Cultures GmbH (DSMZ) (5), which we ordered in 2015 and preserved in our laboratory. The whole-genome sequencing of this strain was performed in 2018. Its other name is *Streptoverticillium* sp. JCM 4673 (6), which was reported to produce 8-azaguanine (7). This strain was grown on GYM streptomyces medium 65 at 28°C and 7.2 pH before the addition of agar. GYM streptomyces medium contained 4.0 g glucose, 4.0 g yeast extract, 10.0 g malt extract, 2.0 g CaCO_3_, and 12.0 g agar. GYM liquid medium without agar is used for mycelium culture.

The culture solution was centrifuged at 5,000 × *g* for 10 min to obtain mycelium. About 20 mg mycelium was put into a 1.5-ml microcentrifuge tube and resuspended with 200 μl Tris-EDTA (TE) buffer. DNA extraction and purification were done using a PureLink microbiome DNA purification kit (Thermo Fisher, USA) following the manufacturer’s instructions. A DNA library was prepared using the MagMAX-96 DNA multisample kit (Thermo Fisher, USA) and protocol (Illumina, CA) and was subsequently paired-end sequenced on an Illumina NextSeq platform using a 2 × 150-bp sequencing kit. Since the original sequencing data of Illumina X Ten had some data of lower quality, in order to make the subsequent assembly more accurate, the original data were quality cut with seqtk trimfq version 1.3 (https://github.com/lh3/seqtk) and TrimGalore version 0.5.0 (https://github.com/FelixKrueger/TrimGalore/releases). *De novo* assembly was carried out using SOAPdenovo version 2.04 (8). Predictions of genes, coding DNA sequences (CDS), and RNA genes were carried out using the NCBI Prokaryotic Genome Annotation Pipeline (PGAP) (9). Tandem repeats were predicted with tandem repeats finder (TRF) software (10). The biosynthetic gene cluster for 8-azaguanine was predicted with antiSMASH version 5.0 (11). All software runs used default parameters.

The *S. morookaense* genome was recovered at 924× coverage as 4,760,411 reads, which were assembled into 238 contigs. The draft genome sequence has an *N*_50_ value of 127,665 bp and a total of 7,469,426 bp with a 71.66% GC content. A total of 6,855 genes were annotated using PGAP and deposited in the GenBank database. Among them, 6,781 CDS consisted of 6,334 protein-coding genes and 447 pseudogenes, and 74 RNA coding genes were responsible for 66 tRNAs, 3 noncoding RNAs (ncRNAs), 1 16S rRNA, and 4 23S (partial) rRNAs. The total length of 943 tandem repeats is 174,279 bp, accounting for 1.70% of the genome.

Streptomyces morookaense DSM 40503 is closely related to Streptomyces luteoverticillatus CGMCC 15060 (GenBank accession number CP034587.1), and their 16S rRNA gene identity was 98.89%. Based on the gene cluster for 8-azaguanine in Streptomyces pathocidini NRRL b-24287 (12), the 8-azaguanine gene cluster was found in Streptomyces morookaense DSM 40503 with the aid of antiSMASH and 2ndFind (http://biosyn.nih.go.jp/2ndfind/). A total of 52 biosynthetic gene clusters involved in the production of different bioactive compounds were predicted in this genome. This work broadened our understanding of the genomes of bacteria producing nitrogen-containing compounds.

### Data availability.

This whole-genome shotgun project has been deposited at DDBJ/ENA/GenBank under the accession number JABBXF000000000. The version described in this paper is version JABBXF000000000.1. The FASTQ files of the raw reads were deposited in the NCBI SRA under accession number PRJNA625712. The 8-azaguanine gene cluster accession number is MT344128.
